# Effect of Manual Tungsten Inert Gas Welding on the Microstructure and Mechanical Properties of Welded Joints in Thick SIMP Steel Plates

**DOI:** 10.3390/ma19142944

**Published:** 2026-07-08

**Authors:** Yunxia Chen, Weiming Zhang, Shanshan Lyu, Jun Dai

**Affiliations:** 1 School of Intelligent Manufacturing and Engineering, Shanghai Polytechnic University, Pudong New Area, Jinhai Road, Shanghai 201209, China; yxchen@sspu.edu.cn (Y.C.); zwm496568522@163.com (W.Z.); 20241510030@sspu.edu.cn (S.L.); 2 School of Materials Science and Engineering, Shanghai Jiao Tong University, Shanghai 200240, China

**Keywords:** SIMP steel, manual tungsten inert gas (TIG) welding, microstructure, mechanical properties

## Abstract

The novel martensitic heat-resistant SIMP steel is considered a primary candidate structural material for Accelerator Driven Sub-critical Systems (ADSs) due to its excellent comprehensive properties. However, welding difficulties arising from its high alloy content represent a major bottleneck for its engineering application. This study explored and established optimized manual Tungsten Inert Gas (TIG) welding process parameters suitable for 20 mm thick SIMP steel plates, involving 24 layers and 110 passes, with an average heat input of approximately 4 kJ/cm, successfully achieving well-formed and defect-free welds. The welded joints were investigated using optical microscopy, scanning electron microscopy, and other performance tests. The results revealed the presence of δ-ferrite and a small amount of brittle Laves phase in the weld metal, leading to uneven hardness distribution. Furthermore, poor interfacial coordination between δ-ferrite and tempered martensite significantly reduced the ductility of the joint. Through optimized process parameters, the impact energy of the weld metal reached 183.9 J, while that of the heat-affected zone was 77.9 J. The room temperature ultimate tensile strength and yield strength of the joint were 802.63 MPa and 596.33 MPa, respectively, meeting the weld performance requirements stipulated in GB/T 38875-2020. This research provides data support for promoting the engineering application of SIMP steel in fields such as nuclear power and for enhancing the long-term service reliability of welded structures.

## 1. Introduction

The continuous growth in global energy demand, coupled with the environmental pollution and climate change issues stemming from over-reliance on traditional fossil fuels, has spurred the search for alternative energy sources. While renewable energies like solar and wind power are becoming increasingly prevalent, their inherent instability and higher costs currently prevent them from fully meeting the reliability demands of energy supply. In this context, nuclear energy, as a clean, stable, and large-scale power source, offers indispensable advantages. Despite challenges related to nuclear waste storage and safety, its long-term role in achieving global carbon emission reduction targets is undeniable [1].

The Accelerator Driven Sub-critical System (ADS) is an innovative nuclear energy application technology. Its structure primarily consists of three parts: a proton accelerator, a heavy metal spallation target, and a sub-critical nuclear reactor. ADS technology enables efficient nuclear waste treatment and energy recovery through neutron transmutation, positioning it as a key solution for sustainable nuclear energy development [2,3]. However, structural materials within ADSs must endure long-term service under extreme conditions, including temperatures ranging from 300 °C to 800 °C, heat flux densities on the order of 10 MW m^−2^, and annual irradiation damage up to 100 dpa (displacements per atom). These demanding conditions impose nearly stringent requirements on the comprehensive performance of materials [4]. Against this backdrop, SIMP steel, a novel martensitic heat-resistant steel for nuclear applications jointly developed by the Institute of Metal Research (IMR), Chinese Academy of Sciences (CAS), and the Institute of Modern Physics (IMP), CAS, has emerged as a leading candidate material. Its favorable attributes include good thermal conductivity, excellent irradiation resistance, a low coefficient of thermal expansion, and superior resistance to liquid metal corrosion, making it the preferred structural material for ADS systems [5]. This aligns with the broader international consensus, where advanced ferritic/martensitic steels have been extensively developed and evaluated as primary structural materials for next-generation reactor concepts [6,7].

Current domestic and international research on welding high-alloy steels for nuclear applications has predominantly focused on modified austenitic stainless steels and Oxide Dispersion Strengthened (ODS) steels [6,7,8]. Systematic research on the welding of SIMP steel, however, is still in its nascent stages. Although SIMP steel exhibits outstanding fundamental properties, its engineering application faces a significant bottleneck: inadequate welding performance. The high concentration of alloying elements in SIMP steel (such as Cr, W, Ta) introduces metallurgical complexities that, during conventional fusion welding processes, can lead to issues such as high susceptibility to weld cracking, grain coarsening in the heat-affected zone (HAZ), and non-uniform distribution of precipitates. These issues result in the mechanical properties, irradiation resistance, and corrosion resistance of the welded joints being significantly inferior to those of the base metal [9]. A more critical issue is the current lack of dedicated welding consumables specifically developed for SIMP steel. Existing commercial filler materials often exhibit mismatches in chemical composition and may not provide the required high-temperature microstructural stability, thereby failing to meet the demands for long-term service reliability of welded structures in ADSs [10]. For instance, a 2022 welding test report from Shanghai Apollo Machinery Co., Ltd. indicated that the impact values for SIMP base material were 50.9 J, 55.8 J, and 56.1 J, with an average of 54.3 J. Another report from Atlantic Welding Materials Co., Ltd. in 2022 on SIMP steel manual TIG clad metal showed a yield strength of 678 MPa, an ultimate tensile strength of 826 MPa, and room temperature impact values of only 11 J, 21 J, and 18 J. This stark contrast, particularly the extremely low impact values, strongly indicates that the availability of a metallurgically compatible and optimized filler material is a primary hurdle. These data reveal that the strength of SIMP steel welds produced with some existing consumables does not meet the national standard requirements for the base material strength. Therefore, there is a pressing need to develop different welding consumables, investigate the microstructure and properties of joints produced by various welding methods, and establish optimized welding processes for SIMP steel. The goal is to ensure that the weld performance is not inferior to that of the base metal, thereby facilitating the broader industrial application of SIMP steel. While this study focuses on process optimization with an existing filler, the results implicitly underscore the urgent need for dedicated research and development into SIMP steel-specific welding consumables.

This study selected a φ400 mm × 1400 mm SIMP steel ingot as the research object. The technical approach involved first preparing welding test plates via forging and testing their room temperature yield strength and tensile strength to ensure compliance with GB/T 38875-2020 standard [11] requirements; secondly, conducting manual TIG welding experiments using filler wire provided by Shanghai Apollo Machinery Co., Ltd. (Shanghai, China); thirdly, systematically evaluating the mechanical properties of the weld (room temperature and high-temperature tensile tests, impact tests) to verify its compliance with standards; and finally, establishing an optimized system of manual TIG welding process parameters. The primary objective of this study is twofold: first, to establish an optimized manual TIG welding procedure and second, to evaluate the evolving microstructural characteristics and their impact on the mechanical properties. In engineering practice, establishing these robust welding techniques directly ensures the long-term operational safety and reliability of heavy-section components in ADS reactors.

## 2. Materials and Experimental Methods

### 2.1. Materials

#### 2.1.1. Base Material (SIMP Steel)

The base material used in this study was a thick SIMP steel plate, forged from a φ400 mm × 1400 mm ingot weighing approximately 1.4 tons. The nominal chemical composition of this SIMP steel is presented in Table 1. Key alloying elements include Cr (10.0–12.0 wt.%), W (1.0–2.0 wt.%), V (0.15–0.30 wt.%), and Si (1.0–2.0 wt.%), with carbon content controlled within 0.14–0.25 wt.%. The remainder is Fe and trace impurity elements (S, P, N, O, etc.).

The forging of the steel plate was performed by Nanjing Puzhen High speed rail Vehicle forging Co., Ltd. (Nanjing, China), following forging process document Q/GT/GC-534-01 [12]. The main forging steps included: sawing the original φ400 mm × 1400 mm ingot into four pieces of φ400 mm × 350 mm; heating these pieces incrementally (furnace temperature set to 1130 °C, heating time of 6 h, with billets loaded when the furnace reached 800 °C) followed by secondary and tertiary forging operations. This process yielded 60 forged plates with dimensions of 28 mm × 158 mm × 608 mm. After inspection confirmed no defects, these plates were further machined into test plates for welding experiments, with final dimensions of 20 mm (thickness) × 150 mm (width) × 600 mm (length).

Room temperature mechanical property tests were conducted on this batch of SIMP steel forged plates. The tensile test results are shown in Table 2. The average 0.2% proof yield strength (Rp0.2) was measured to be 355.6 MPa, and the average ultimate tensile strength (Rm) was 623.6 MPa. Room temperature Charpy V-notch impact test results are presented in Table 3, with an average absorbed energy (KV2) of 88.1 J.

A comparison of the measured mechanical properties of the SIMP steel forged plates with the minimum requirements for room temperature (20 °C) performance specified in the national standard GB/T 38875-2020 (Table 4) reveals that the forged plates used in this study exhibited lower yield strength, tensile strength, and impact toughness than the standard’s minimums for delivered plates. This discrepancy is primarily because the GB/T 38875-2020 standard typically pertains to rolled plates that have undergone specific heat treatments, and rolled products generally exhibit higher mechanical properties than forged products due to differences in processing methods and resulting microstructures. The initial mechanical state of the as-received forged SIMP steel (average Yield Strength (YS) 355.6 MPa, Ultimate Tensile Strength (UTS) 623.6 MPa, impact energy 88.1 J) being lower than the standard for heat-treated rolled plates (YS ≥ 550 MPa, UTS ≥ 800 MPa, impact ≥ 120 J) underscores that the base material itself might require further optimization. However, the impact toughness of this batch of forged base material (88.1 J) was relatively higher than the average value of 54.3 J reported in the welding test report by Shanghai Apollo Machinery Co., Ltd. (Shanghai, China) for their SIMP base material, suggesting potential variability in base material processing or quality. The effect of post-weld heat treatment on martensitic heat-resistant steel weldments is a relevant consideration [13,14,15].

#### 2.1.2. Welding Consumables

The welding wire used in this study was supplied by Shanghai Apollo Machinery Co., Ltd. (Shanghai, China). Its chemical composition is detailed in Table 5. The chemical composition of the filler wire, particularly its chromium content (approximately 9.0 wt.%), is lower than that of the SIMP base material (10.0–12.0 wt.% Cr). This under-matching in chromium, a ferrite stabilizer, is anticipated to influence the phase balance in the weld metal. Conversely, the manganese content in the filler wire (0.93–1.06 wt.%) is notably higher than that typically found in the base metal (BM Mn ≤ 0.27 wt.% from subsequent EDS analysis). These compositional differences are expected to directly impact the weld pool chemistry and subsequent phase transformations.

### 2.2. Welding Procedure

Manual Tungsten Inert Gas (TIG) welding was employed in this study to weld the thick SIMP steel plates. The welded joint was designed as a butt joint of two SIMP steel plates, each with dimensions of 600 mm in length, 150 mm in width, and 20 mm in thickness. A single V-groove configuration was adopted for the joint preparation, with a groove angle of 30° on each side, resulting in a total included angle of 60° upon assembly. The root face height was set to 1 mm, and the assembly gap between the two test plates was 2.5 mm. Prior to welding, all test plates were preheated to 200 °C. The welding was performed in a multi-layer and multi-pass manner, consisting of 24 layers and 110 passes. The welding sequence started from the root pass and then proceeded successively through the filling passes to the cover passes. The detailed joint geometry and welding arrangement are summarized in Table 6.

The TIG welding process used a filler wire of φ1.2 mm. High-purity argon (99.99%) was used as the shielding gas. For the root pass, back-purging was implemented on the reverse side of the weld to prevent oxidation and ensure proper root penetration and formation. Specific welding process parameters are detailed in Table 7. During the welding process, surface oxides and slag on interpass and interlayer weld beads were thoroughly removed using stainless steel grinding tools or wire brushes to ensure weld quality.

To achieve good welded joint properties, this procedure stipulated strict control over preheating, interpass temperature, and post-weld heat treatment (PWHT). Before welding, the groove area and adjacent base material were preheated to 200 °C. During multi-layer welding, the interpass temperature was also controlled to be within 200 °C. This control is critical for martensitic steels to manage cooling rates of subsequent passes and avoid over-tempering or uncontrolled re-austenitization of prior layers. After welding completion, the joint underwent a two-stage heat treatment:

Post-heating: The welded assembly was air-cooled to 80 °C to 90 °C and held for 1 h. Subsequently, it was heated at a rate of 200 °C h^−1^ to 300 °C and held for 2 h. This was followed by air cooling to room temperature and resting for 24 h. This initial low-temperature treatment primarily serves as a hydrogen diffusion treatment to mitigate cold cracking risks. PWHT (Tempering/Stress Relief): After the 24 h rest, the component was furnace-heated at 200 °C h^−1^ to 720 °C (with a 20 min hold at 600 °C during ramp-up). It was held at 720 °C for 4 h. After the holding period, it was air-cooled to room temperature. This higher-temperature stage is for tempering the as-welded martensite to improve toughness and ductility, and for relieving residual stresses.

### 2.3. Experimental Methods

**Figure 1:**
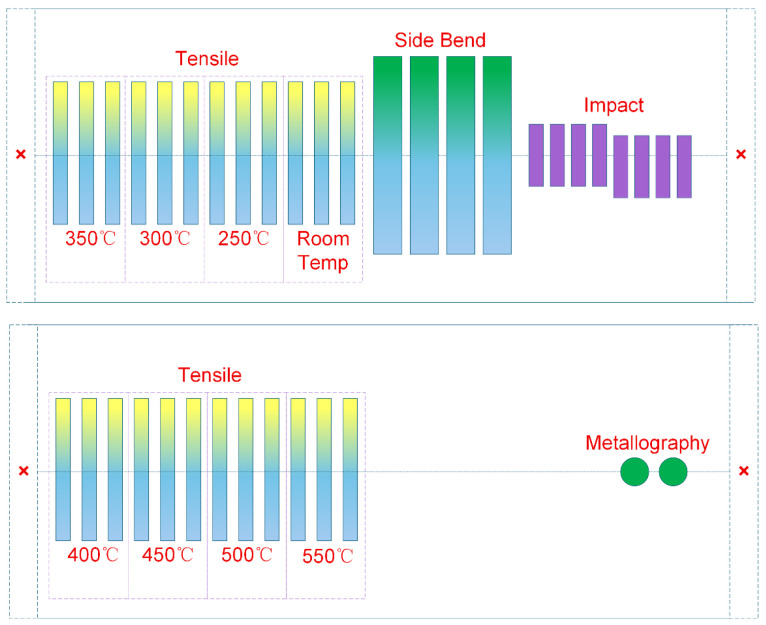
Schematic Diagram of Welding Specimen Sampling (600 × 150 × 20 mm, 2 pieces, with 20 mm cut off from each end of the welded specimens).

To comprehensively evaluate the effect of welding process parameters on the properties of the welded joint, systematic non-destructive testing (NDT), microstructural observation, and mechanical property testing were conducted on the welded test blocks. NDT, including visual inspection (VT) and radiographic testing (RT) according to ASME-2004-V standards [16], was performed first. Qualified weldments were then used for the preparation of various test specimens. As schematically detailed in Figure 1, the test specimens were sequentially extracted from two 600 mm long welded test plates. To avoid arc start and crater effects, 20 mm of material was discarded from both ends of each plate. From the first plate (Figure 1, top), specimens for tensile testing at 350 °C, 300 °C, 250 °C, and room temperature were extracted from the left section, followed sequentially by four side bend test specimens, and finally the impact test specimens from the right section. From the second plate (Figure 1, bottom), high-temperature tensile specimens (for testing at 400 °C, 450 °C, 500 °C, and 550 °C) were machined from the left section, while the full cross-section specimens for macro/micro-metallography and hardness testing were extracted from the remaining right section. Figure 1 The test temperatures, number of samples, sampling locations, and test methods for each evaluation item are summarized in Table 8. The consistent application of recognized standards (ASME, ASTM, AWS) ensures the reliability and comparability of the results.

For metallographic analysis, samples were prepared using wire electrical diskharge machining (WEDM) to extract full cross-section specimens, ensuring coverage of the weld metal (WM), heat-affected zone (HAZ), and base metal (BM). After sectioning via WEDM and mounting, the samples underwent standard grinding and polishing procedures using an M-2A dual-disc metallographic pregrinding machine (Shanghai Precision Instruments Co., Ltd., Shanghai, China).Prior to the final microscopic examination, the polished surfaces were cleaned and chemically etched. Microstructural observations were conducted according to ASTM E3 [17], E340 [18], and E407 [19] standards. A Zeiss Imager A2 optical microscope (Carl Zeiss AG, Oberkochen, Germany) was used for general microstructural analysis, while a JSM-7600F scanning electron microscope (FE-SEM, JEOL Ltd., Tokyo, Japan) equipped with an Energy Dispersive X-ray Spectrometer (EDS) was employed for more detailed examination of morphology, precipitate analysis, and elemental distribution [20]. This multi-scale characterization approach, combined with localized EDS and comprehensive mechanical testing, allows for strong structure-property correlations.

**Table 8:** Mechanical Property Testing Plan.

Test Item	Test Temperature	No. of Samples/Location	Test Method
Joint Tensile	Room Temp.	3, Perpendicular to welding direction	AWS B4.0M [21]
	250 °C	3, Perpendicular to welding direction	ASTM E21 [22]
	300 °C	3, Perpendicular to welding direction	ASTM E21
	350 °C	3, Perpendicular to welding direction	ASTM E21
	400 °C	3, Perpendicular to welding direction	ASTM E21
	450 °C	3, Perpendicular to welding direction	ASTM E21
	500 °C	3, Perpendicular to welding direction	ASTM E21
	550 °C	3, Perpendicular to welding direction	ASTM E21
Weld Metal Impact	Room Temp.	1 group of 4, Notch axis at weld center	AWS B4.0M
HAZ Impact	Room Temp.	1 group of 4, Notch axis 2 mm from fusion line	AWS B4.0M
Side Bend	Room Temp.	4, Perpendicular to welding direction	AWS B4.0M
UT Standard Block	/	1	/
Macro Metallography	Room Temp.	1, Full cross-section specimen	ASTM E3, E340
Micro Metallography	Room Temp.	1, Full cross-section specimen	ASTM E3, E407
Hardness	Room Temp.	1, Full cross-section specimen	AWS B4.0M

Mechanical property tests included tensile, bending, and impact tests. According to ASTM E21 and standard modifications, cylindrical tensile specimens were machined with a gauge diameter of approximately 6.35 mm (0.25 inch) and M12 × 1.75 threaded ends. These tensile specimens were extracted perpendicular to the welding direction and tested using a Zwick Z100 static tensile testing machine (Zwick, Ulm, Germany) at room temperature and elevated temperatures (ranging from 250 °C to 550 °C), with three specimens tested at each temperature. Side bend tests were performed on a bend testing machine to evaluate the bending deformation capability of the welded joint. Impact tests were conducted at room temperature in strict accordance with the AWS B4.0M standard using standard Charpy V-notch specimens (dimensions: 55 mm × 10 mm × 10 mm, with a 45° V-notch of 2 mm depth and 0.25 mm root radius). The specific configuration involved testing the impact toughness of the weld metal (with the notch axis exactly at the weld center) and the HAZ (with the notch axis located 2 mm from the fusion line), with one group of four specimens tested for each location. Hardness testing was performed on full cross-section specimens along a line perpendicular to the weld, using a Zwick ZHV zwicki-Line 30 automatic Vickers hardness tester (Zwick, Ulm, Germany), in accordance with AWS B4.0M, to obtain hardness profiles across the WM, HAZ, and BM.

## 3. Results and Discussion

### 3.1. Microstructure of the Welded Joint

**Figure 2:**
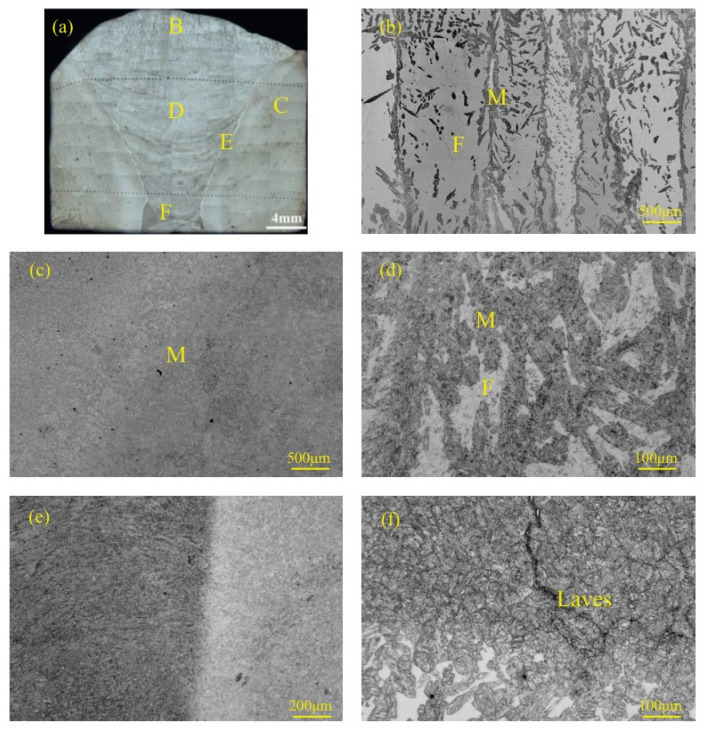
Microstructural features and evolution across different regions of the welded joint: (**a**) Macrograph of the welded joint showing sampling locations; (**b**) Microstructure of the weld top region; (**c**) Microstructure of the base metal (BM) region; (**d**) Microstructure of the weld middle region; (**e**) Microstructure of the fusion line region; (**f**) Microstructure of the weld bottom region. (B, C, D, E, and F in (**a**) denote the sampling locations corresponding to subfigures (**b**–**f**); M and F denote martensite and δ-ferrite, respectively.)

Figure 2a shows a macrograph of the welded joint and indicates the locations for microstructural observation, which primarily included the top, middle, and bottom of the weld, the region near the fusion line, and the base metal zone. The microstructure of the base metal (BM) region (Figure 2c) was uniform, characterized by finely dispersed particles, identified as tempered martensite, which imparts high strength to the material. This BM microstructure was affected by the heat input during welding, leading to property changes in the HAZ.

The weld metal (WM) predominantly consisted of martensite with some δ-ferrite. The V-groove geometry and multi-pass nature of the welding process resulted in a highly non-uniform microstructure within the WM. Different regions experienced varying cooling rates and degrees of elemental segregation, which significantly influenced the formation and morphology of δ-ferrite [23]. The V-groove design inherently leads to a gradient in thermal experience from the root to the cap, directly explaining these observed variations. In the weld top region (Figure 2b), where the volume of deposited metal was largest and heat dissipation was slower, the cooling rate was relatively low. This allowed more time for the nucleation and growth of δ-ferrite, resulting in a higher content of this phase, which appeared with a dendritic morphology within the martensitic matrix. The weld middle region (Figure 2d) also exhibited a mixed microstructure of martensite and δ-ferrite, but the ferrite morphology differed from that at the top, appearing as reticular or granular networks. Near the fusion line (Figure 2e), the left side of which shows the WM, good fusion was observed. This region contained the least amount of filler metal per unit volume and experienced rapid heat dissipation to the cooler base metal, leading to high cooling rates. Consequently, δ-ferrite formation was suppressed, its content was lower, and the overall microstructure (likely dominated by lath martensite) appeared more lath-like. It should be noted that due to the complex thermal cycles induced by the 110 welding passes, the HAZ near the fusion line exhibits a highly inhomogeneous microstructural gradient. The repetitive heating and non-equilibrium cooling processes result in significant microstructural blending, making the boundaries between distinct phases and grain structures difficult to distinguish sharply under standard optical and scanning electron microscopy. Notably, in the weld bottom region (Figure 2f), a small amount of brittle Laves phase was observed, distributed along grain boundaries. The presence of Laves phase, even in small quantities, particularly at grain boundaries in the weld root (a high-stress concentration area), is a concern as it can be detrimental to toughness and ductility, potentially serving as sites for crack initiation [24].

### 3.2. EDS Analysis of the Welded Joint

**Figure 3:**
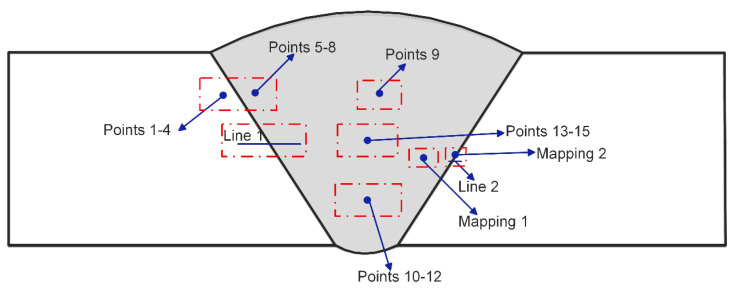
Schematic diagram showing key regions selected for EDS analysis in the welded joint.

Figure 3 shows a schematic of the key regions selected for Energy Dispersive X-ray Spectroscopy (EDS) analysis on the welded joint. Line 1 traversed the weld pool from the base metal region to the weld center, while Line 2 was positioned along the direction of the maximum thermal gradient. Mapping 1 in the weld zone was used to examine the types and distribution of elements within the weld, and Mapping 2 of the fusion line was performed at the weld edge to observe compositional changes induced by high welding temperatures.

**Figure 4:**
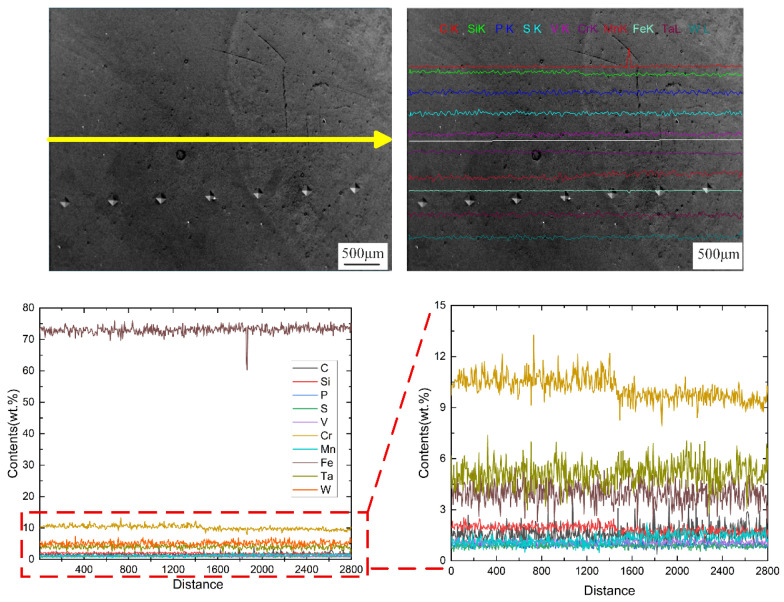
EDS line scan results (Line 1) near the fusion line.

**Figure 5:**
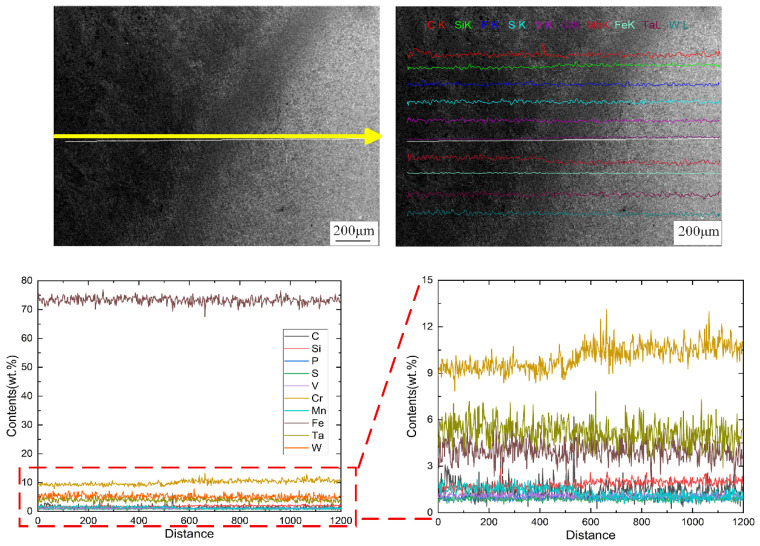
EDS line scan results (Line 2) near the fusion line.

Figure 4 and Figure 5 present the low and high magnification line scan results near the weld fusion line. The line scan results indicate that there were differences in elemental distribution between the base metal and weld metal regions, particularly for Cr and Si. Magnified views of the line scan results showed that while the distribution of various elements within each respective zone (BM or WM) was relatively consistent, the Cr content in the base metal was distinctly higher than in the weld metal. Similarly, the Si content in the base metal region was also higher than in the weld zone. These differences are likely attributable to the influence of welding heat input or cooling conditions on elemental migration and dilution from the filler metal.

**Figure 6:**
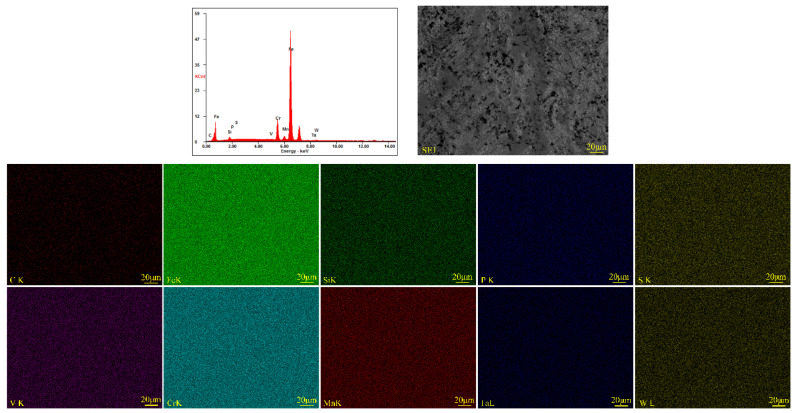
EDS elemental mapping results (Mapping 1) near the weld zone.

**Figure 7:**
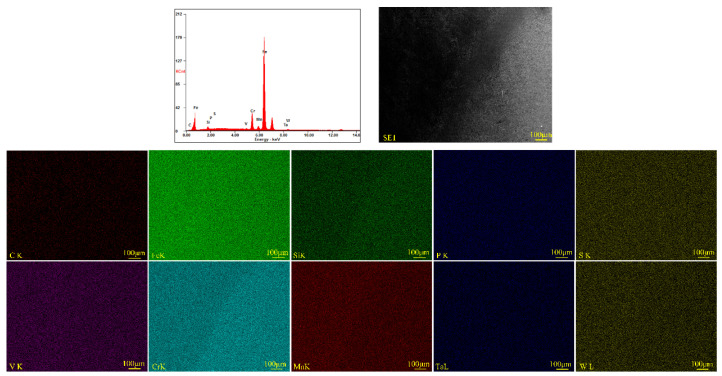
EDS elemental mapping results (Mapping 2) near the fusion line.

Figure 6 and Figure 7 present the low and high magnification EDS elemental mapping results near the weld zone and fusion line, respectively. The elemental distribution appears relatively uniform, with no significant segregation observed. However, subtle differences in the distribution of Si and Cr are noticeable between the weld metal and base metal regions, aligning with the elemental distribution trends identified through EDS line scans and point analyses.

As indicated in Figure 3, these points included the base metal region near the fusion line (Points 1–4), the weld metal region near the fusion line (Points 5–8), and the weld center region (Points 9–15). The detailed elemental composition (wt.%) for each point is provided in Table 9.

The EDS results in Table 9 confirm significant compositional differences between the base metal (Points 1–4) and the weld metal (Points 5–15). The BM exhibited markedly higher Cr (10.40–10.72 wt.%) and Si (1.62–1.79 wt.%) contents compared to the WM. These quantitative results are consistent with the nominal compositions of the BM and the Apollo filler wire, where the filler has lower Cr. Conversely, elements such as V, Mn, and W were generally found in higher concentrations in the WM. Vanadium content was slightly higher in the WM (0.30–0.34 wt.%) than in the BM (0.25–0.27 wt.%). Tungsten content was also higher in the WM (1.92–2.23 wt.%) compared to the BM (1.46–1.61 wt.%). Notably, manganese content was significantly higher in the WM (0.59–0.75 wt.%) than in the BM (0.10–0.27 wt.%), reflecting the higher Mn content of the filler wire. Further examination of the compositional distribution within the WM (Points 5–15) revealed that moving from the region near the fusion line (Points 5–8) towards the weld center (Points 9–15), the Cr and Si contents showed a slight decreasing trend, while V, Mn, and W contents exhibited a slight increasing trend. These gradients suggest complex solidification and diffusion phenomena, possibly related to varying degrees of dilution from the base metal with distance from the fusion line and elemental diffusion behavior during the multiple thermal cycles of welding. Such micro-segregation can lead to localized variations in phase stability and properties [25].

### 3.3. Microhardness of the Welded Joint

**Figure 8:**
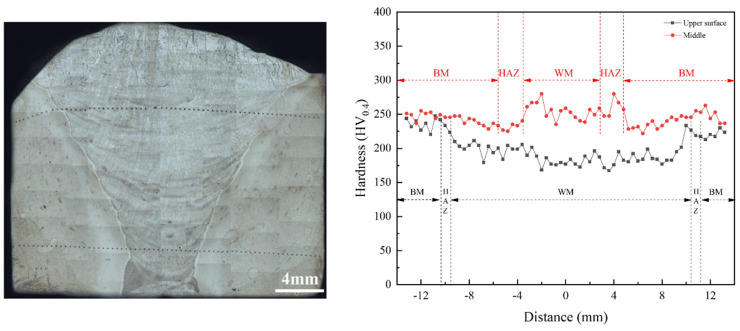
Microhardness distribution across the welded joint cross-section.

Figure 8 shows the Vickers microhardness distribution across the cross-section of the manually TIG welded joint [26]. Measurements (HV0.4) were taken along lines located 2 mm below the top surface of the weld and 2 mm above the weld root, with the *x*-axis representing the distance from the weld center.

The results indicate that the hardness of the base metal (BM) was relatively stable. In the weld top region (measurements 2 mm from the surface), the BM hardness was maintained around 230 HV, the HAZ hardness was also approximately 230 HV, while the weld metal (WM) hardness was generally lower than the BM, around 190 HV. In contrast, for the weld root region (measurements 2 mm from the root surface), the BM hardness was about 250 HV, the HAZ hardness was around 240 HV, and the WM hardness reached its peak, approximately 250 HV.

From the cross-sectional microhardness results, it can be observed that the hardness near the top surface was lower than that at the root position, with a noticeable decrease in hardness near the weld center in the top region. The hardness fluctuations in the HAZ are likely related to microstructural transformations and stress concentrations caused by the welding thermal cycles. The lower hardness of the WM near the top surface compared to the BM may be associated with the composition, microstructure (e.g., higher δ-ferrite content as seen in Figure 2b), and cooling rate of the weld metal in that region. The overall lower hardness at the top surface could also be influenced by oxidation during welding, specific cooling conditions, and residual stress distribution. The significant hardness drop in the top region of the WM (190 HV) compared to the BM (230 HV) creates a “soft zone,” which can sometimes be a site for strain localization. The harder WM root matching the BM hardness is generally favorable for load transfer, provided it is not brittle [27].

### 3.4. Tensile Properties of the Welded Joint

#### 3.4.1. Tensile Test Results

Table 10 summarizes the tensile test results for the SIMP steel manual TIG welded joints, conducted at temperatures ranging from room temperature to 550 °C. The table records key mechanical property indicators including ultimate tensile strength (Rm), yield strength (Rp0.2), elongation, and fracture location [28].

In this study, the tensile behavior was evaluated using the 0.2% proof strength Rp0.2, ultimate tensile strength Rm, elongation, and fracture location. Since no distinct upper or lower yield point was reported in the tensile test results, Rp0.2 was adopted as the yield-strength parameter instead of Re, ReH, or ReL.

The results show that as the test temperature increased, both the ultimate tensile strength and yield strength gradually decreased, while the elongation gradually increased. Notably, fracture consistently occurred in the weld metal (WM) region across all test temperatures. Furthermore, it was observed that at 450 °C and 550 °C, the grain boundary strength was significantly reduced.

The consistent fracture in the WM, coupled with microstructural observations (Section 3.1), suggests that δ-ferrite, present in the WM, plays a critical role. The macro-metallography of the welded joint indicated that the WM contained a certain amount of δ-ferrite, which has a lower microhardness (approximately 170 HV) compared to the high-temperature tempered martensite in the base material (around 230–250 HV, or even interpreted as 300 HV in some contexts). This disparity contributes to the relatively lower yield strength and tensile strength of the weld metal. Moreover, an increase in δ-ferrite content leads to a greater number of interfaces between the δ-ferrite and the tempered martensite. The poor deformation compatibility at these two-phase interfaces can easily induce local stress concentrations during tensile loading, promoting rapid necking and unstable fracture, thereby reducing ductility. The observation of significantly reduced grain boundary strength at 450 °C and 550 °C is critical for high-temperature applications, potentially indicating a shift towards intergranular failure mechanisms or accelerated creep due to phenomena like grain boundary sliding or deleterious segregation/precipitation [29].

#### 3.4.2. Fractography Analysis of Tensile Specimens

**Figure 9:**
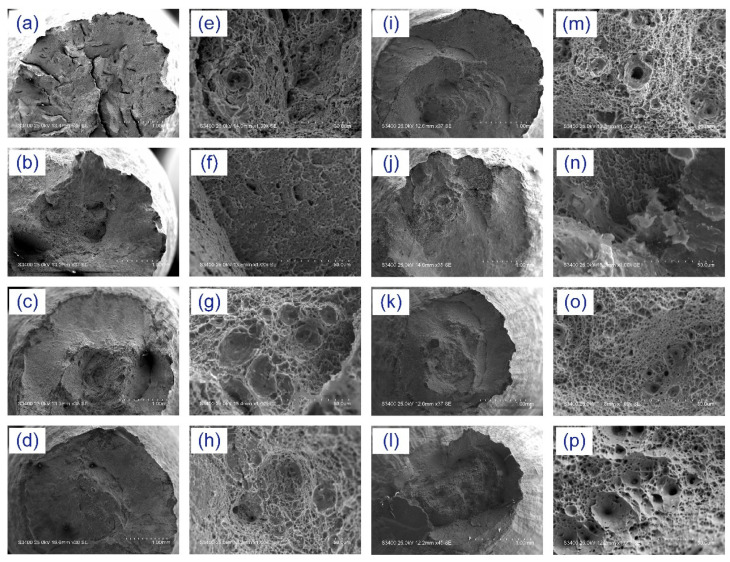
SEM fractographs of tensile specimens tested at different temperatures: (**a**,**e**) Room temperature; (**b**,**f**) 250 °C; (**c**,**g**) 300 °C; (**d**,**h**) 350 °C; (**i**,**m**) 400 °C; (**j**,**n**) 450 °C; (**k**,**o**) 500 °C; (**l**,**p**) 550 °C.

Analysis of the fracture surfaces of tensile specimens tested at different temperatures (Figure 9) revealed that the overall fracture exhibited typical cup-cone characteristics, primarily composed of a fibrous zone and a radial zone, with only a minimal shear lip. The fibrous zone on the fracture surface was covered with a large number of micron-scale and nano-scale dimples. The presence of fine, small dimples between larger dimples indicates that the SIMP steel welded joint possesses good ductile fracture characteristics [30].

From room temperature to 550 °C, the micro-morphology of the fracture surface was consistently composed of shear-type shallow dimples and equiaxed dimples, but their relative proportions changed significantly with temperature. In the temperature range from room temperature to 400 °C, the fracture surface was predominantly characterized by shear-type dimples. Delamination cracks observed were deeper and more widely spaced, reflecting that the material primarily underwent shear plastic deformation in this temperature range, exhibiting higher strength. These “delamination cracks” might be linked to decohesion at interfaces between δ-ferrite and martensite, stemming from their poor deformation compatibility. When the temperature was increased to 450 °C, the proportion of shear dimples significantly decreased, while the number of equiaxed dimples increased, and their undulation became more pronounced. At this temperature, the material’s strength decreased, but its elongation remained stable. This change is possibly due to the increased temperature promoting dislocation mobility, leading to more uniform plastic deformation. At the high temperature of 550 °C, the equiaxed dimples became larger and deeper, corresponding to a further decrease in the material’s strength and a significant increase in elongation. This indicates that the material’s plastic deformation capability was markedly enhanced at high temperatures, with the fracture mode being predominantly ductile. Despite changes in dimple morphology, the fracture remained ductile across the tested temperature range, which is a positive sign for structural reliability.

### 3.5. Room Temperature Impact Toughness

#### 3.5.1. Impact Test Results

The impact performance of a welded joint is a key indicator for evaluating the comprehensive mechanical properties of welded structures, as its value directly reflects the joint’s ability to resist fracture under dynamic loading [31]. In this study, to comprehensively assess the impact toughness of the welded joint, systematic tests were conducted specifically on the weld metal (WM) and the heat-affected zone (HAZ). Standard Charpy V-notch impact specimens with a thickness of 10 mm were used, and sampling and testing were performed in strict accordance with the impact test requirements of the international welding standard AWS B4.0M:2000 (R2010).

Table 11 details the raw data from the room temperature impact tests. For the manual TIG welded specimens, three valid impact energy values were obtained for the weld metal: 177.7 J, 181.7 J, and 192.2 J (two other data points were discarded due to specimen preparation issues). For the heat-affected zone, five valid data points were obtained: 67.1 J, 77.4 J, 66.1 J, 92.0 J, and 87.1 J.

Analysis of these results shows that the average impact energy for the weld metal reached 183.9 J, which is significantly higher than the minimum requirement of 120 J specified in GB/T 38875-2020 for the base material. The excellent impact toughness of the WM, despite the presence of δ-ferrite, suggests that the morphology and distribution of this phase, along with the benefits of PWHT, were favorable. All individual impact energy values for the weld metal specimens exceeded 170 J, and the data exhibited low scatter (range of only 14.5 J), indicating excellent and stable impact toughness in the weld zone. In contrast, the average impact energy for the heat-affected zone was 77.9 J. Although lower than that of the weld metal, it remained at a certain level. However, the HAZ impact performance showed greater variability (range of 25.9 J), which may be related to microstructural inhomogeneity in this region. The significantly lower average impact toughness of the HAZ clearly identifies it as a potential weak zone in terms of dynamic fracture resistance. Based on the comprehensive test data, it can be clearly determined that the weld metal of this manual TIG welded joint fully meets the technical requirement of impact energy not less than 120 J. Its excellent impact performance provides a reliable guarantee for the safe service of welded structures under dynamic loading conditions.

#### 3.5.2. Impact Fractography Analysis

**Figure 10:**
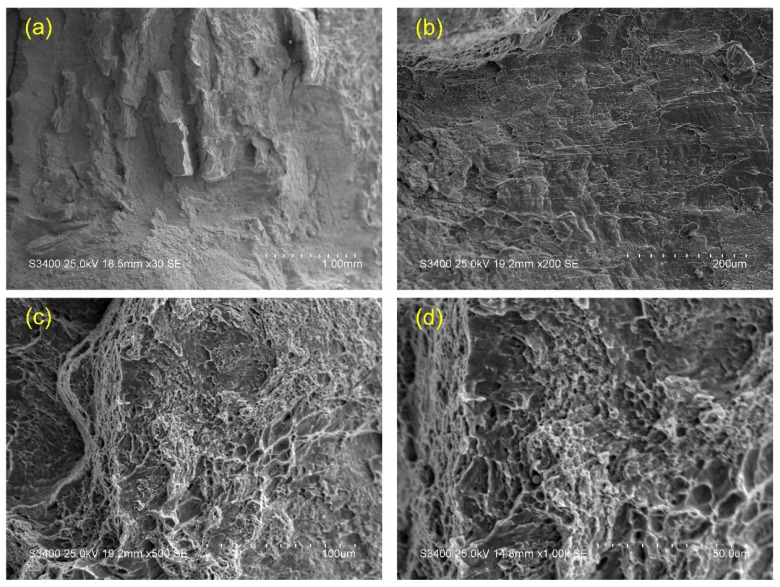
Impact fracture surface morphology of the weld metal (WM) region: (**a**,**b**) Low magnification; (**c**,**d**) High magnification showing details of dimples.

**Figure 11:**
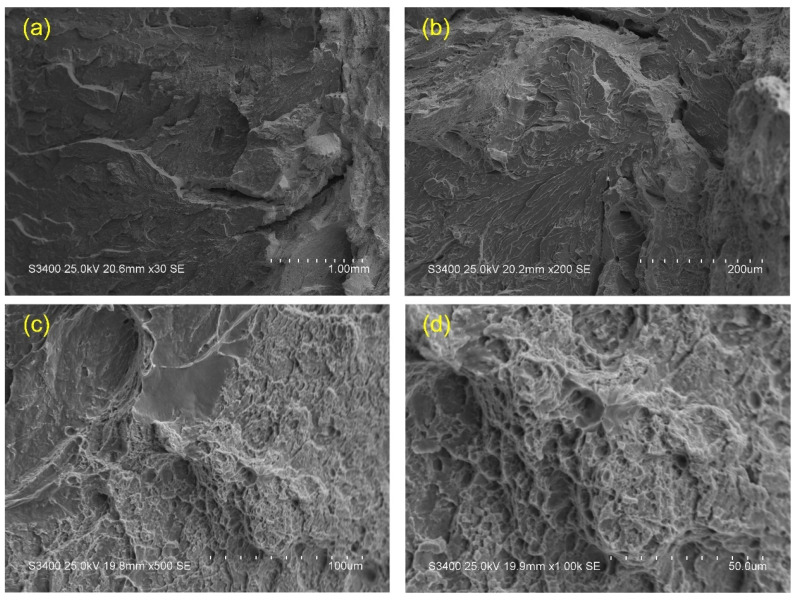
Impact fracture surface morphology of the heat-affected zone (HAZ) region: (**a**,**b**) Low magnification showing cleavage facets; (**c**,**d**) High magnification showing mixed cleavage and dimples.

To gain a deeper understanding of the performance differences exhibited by the welded joint during impact testing, the micro-morphology of the impact fracture surfaces was examined in detail using scanning electron microscopy (SEM). The results for the weld metal (WM) region are shown in Figure 10, and for the heat-affected zone (HAZ) in Figure 11.

Figure 10 displays the impact fracture morphology of the WM region. Low magnification images (Figure 10a,b) reveal that the fracture surface was significantly rough and irregular, with clear macroscopic evidence of plastic deformation, indicative of good toughness.

At higher magnifications (Figure 10c,d), a large number of deep and uniformly distributed dimples, typically ranging from 5 to 15 μm in diameter based on scale bar estimation, are clearly visible. These dimples are characteristic features of a micro-void coalescence (MVC) fracture mechanism, indicating that the material underwent significant plastic deformation before fracturing. The formation of dimples results from the nucleation of micro-voids at inclusions or second-phase particles, followed by their growth and linkage during plastic deformation. The shape of these dimples is typically equiaxed or elliptical, and their depth and density directly reflect the material’s capacity to absorb energy [32]. This fracture mode, dominated by dimples, strongly indicates that the weld metal possesses excellent plastic deformation capability and high impact toughness, which is highly consistent with the measured high impact energy (average 183.9 J) and further confirms the weld zone’s good resistance to brittle fracture.

In contrast, Figure 11 shows the impact fracture morphology of the HAZ region. Distinctly different from the ductile fracture features of the WM, the HAZ fracture surface exhibited a flatter appearance with multi-scale features indicative of a mixed fracture mode. Low magnification images (Figure 11a,b) clearly show prominent cleavage facets. These facets are smooth surfaces often accompanied by river patterns, which are typical characteristics of brittle cleavage fracture, indicating that cracks propagated rapidly along specific crystallographic planes with minimal plastic deformation [33]. At higher magnifications (Figure 11c,d), although some shallow and small dimples can be observed, their number and depth are far less than in the WM region. These dimples often coexist with brittle fracture areas, forming a mixed cleavage-dimple mode [34,35]. This fracture mode, predominantly characterized by brittle cleavage, indicates that the HAZ exhibited lower plastic deformation capability and resistance to crack propagation under impact loading, which directly corroborates its lower measured impact energy (average 77.9 J). The propensity for brittle behavior in the HAZ is likely related to microstructural changes such as grain coarsening induced by the welding thermal cycle. This highlights that even with optimized PWHT, some HAZ regions may not achieve desired toughness.

### 3.6. Bend Test of the Welded Joint

**Figure 12:**
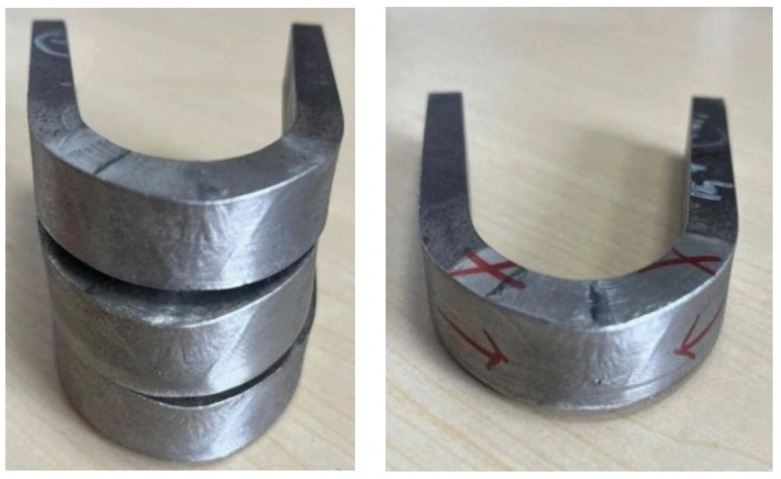
Welded joint side bend test specimens after 180° bending.

In accordance with the AWS B4.0M-2000 (R2010) standard, four side bend test specimens from the welded joint were subjected to a 180° bend test, as shown in Figure 12. Side bend tests are particularly effective for evaluating the overall performance and crack resistance of the weld and adjacent regions along the thickness direction, as they impose uniform tensile stress across the entire specimen thickness, forcing the material to undergo plastic deformation during bending.

According to the test results, three of the welded joint specimens, after bending, showed no visible cracks, tears, or significant defects in the bent region. This indicates that these specimens, representing the welded joint, possessed good ductility and soundness, capable of withstanding severe plastic deformation without fracturing. However, in the remaining one specimen, although its macroscopic appearance after bending was similar to that of the specimens that passed the test, a distinct crack approximately 2 mm to 3 mm in length was observed on the tension side of the bend. This failure may be related to the presence of a small amount of brittle Laves phase in some areas of the weld zone, as noted in the microstructural analysis (Figure 2f). This highlights that localized inconsistencies, such as the presence of Laves phase at the weld root, can impact bulk performance under severe deformation, even if the overall process produces generally sound welds.

## 4. Conclusions

This study established an optimized manual TIG welding procedure for 20 mm thick SIMP steel plates. The main conclusions are summarized as follows:

Optimized Process: Implementing a 200 °C preheat and interpass temperature, coupled with a specialized two-stage post-weld heat treatment, successfully achieved well-formed joints with good overall property control.

Excellent Weld Metal (WM) Toughness: The room temperature ultimate tensile strength of the joint reached 802.63 MPa. Benefiting from a tempered martensite matrix, the WM demonstrated superior impact toughness (183.9 J), exhibiting a completely ductile fracture mode and far exceeding the base metal standard.

HAZ Degradation: The welding thermal cycle induced significant grain coarsening in the heat-affected zone (HAZ), reducing its impact toughness to 77.9 J. Characterized by a mixed-mode fracture, the HAZ constitutes the weakest link in the joint’s dynamic fracture resistance.

Microstructural Heterogeneity: The presence of brittle Laves phase at the weld root (causing local hardness peaks up to 250 HV) and δ-ferrite within the WM limited the tensile ductility and bend test performance due to poor interfacial compatibility.

## Figures and Tables

**Table 1:** Chemical Composition of SIMP Steel (wt.%).

Element	Content (wt.%)	Element	Content (wt.%)
C	0.14–0.25	Co	≤0.01
Cr	10.0–12.0	Sn	≤0.005
Si	1.0–2.0	Mo	≤0.005
W	1.0–2.0	As	≤0.005
Mn	≤1.0	Sb	≤0.005
V	0.15–0.30	Ag	≤0.005
Ta	0.1–0.2	Nb	≤0.005
B	≤0.02	S	≤0.005
Ni	≤0.01	P	≤0.005
Al	≤0.01	N	≤0.005
Ti	≤0.01	O	≤0.003
Cu	≤0.01	Fe	Bal.

**Table 2:** Room Temperature Tensile Test Results of SIMP Steel Forged Plate.

Specimen ID	Yield Strength Rp0.2 (MPa)	Avg. Yield Strength Rp0.2 (MPa)	Tensile Strength Rm (MPa)	Avg. Tensile Strength Rm (MPa)	Elongation (%)
1	351.5	355.6	617.6	623.6	23.4
2	359.7		629.5		23.7

**Table 3:** Room Temperature Impact Test Results of SIMP Steel Forged Plate.

Specimen ID	Impact Absorbed Energy KV2 (J)	Avg. Impact Absorbed Energy KV2 (J)
1	82.4	88.1
2	86.1	
3	71.4	
4	118.8	

**Table 4:** Mechanical Properties of Steel Plate in Delivery Condition (GB/T 38875-2020).

Property	Value
Grade	20Cr11W2VTaSi
Code	SIMP
Recommended Heat Treatment	1000 °C~1100 °C Air cool after soaking; then 720 °C~800 °C Air cool after soaking.
Specified Proof Strength (Rp0.2/MPa, not less than)	
20 °C	550
450 °C	420
550 °C	320
600 °C	230
Tensile Strength (Rm/MPa, not less than)	
20 °C	800
450 °C	500
550 °C	400
600 °C	320
Impact Absorption Energy ^a^ (KV2/J, not less than)	
20 °C	120
−20 °C	30

^a^ Average of three specimens. A single value from one specimen is allowed to be lower than the specified value, but not less than 70% of it.

**Table 5:** Chemical Composition of Welding Wire (wt.%).

Source	C	Cr	W	As	Mn	Si	P	S	Cu	Ta
Apollo wire	0.11	9.04	1.94	0.089	1.06	0.497	0.009	0.0037	0.0046	0.162
Cladding metal	0.12	8.99	2.15	<0.0015	0.93	0.5	<0.0005	0.003	0.0056	0.153

**Table 6:** Geometry parameters and welding arrangement of the V-groove joint.

Item	Description/Value
Joint type	Butt joint
Plate material	SIMP steel
Plate size	600 mm × 150 mm × 20 mm
Plate thickness	20 mm
Groove type	Single V-groove
Groove angle	60° total included angle, 30° on each side
Root face	1 mm
Assembly gap	2.5 mm
Welding process	Manual TIG welding
Welding arrangement	24 layers and 110 passes
Welding sequence	From root pass to filling passes and cover passes

**Table 7:** Manual TIG Welding Process Parameters.

Filler Wire Dia.	Current (A)	Voltage (V)	Shielding Gas Flow Rate (L/min)	Welding Speed (cm/min)
φ1.2 mm	95–105	10–12	Face: 8–12 Back: 6–12	9–10

**Table 9:** EDS Analysis Results for Various Points in the Weld Zone (wt.%).

Point	C	Si	V	Cr	Mn	Ta	W	Fe
1	1.35	1.62	0.25	10.40	0.27	0.50	1.61	Bal.
2	1.35	1.79	0.25	10.72	0.10	0.54	1.49	Bal.
3	1.36	1.78	0.27	10.71	0.15	0.55	1.50	Bal.
4	1.28	1.73	0.25	10.58	0.15	0.51	1.46	Bal.
5	1.41	1.44	0.29	9.74	0.49	0.51	1.81	Bal.
6	1.93	1.42	0.29	9.72	0.51	0.38	1.73	Bal.
7	1.00	1.36	0.29	9.58	0.54	0.51	1.90	Bal.
8	1.03	1.30	0.31	9.40	0.64	0.38	1.96	Bal.
9	1.25	1.30	0.31	9.26	0.70	0.45	2.14	Bal.
10	1.35	1.31	0.32	9.17	0.69	0.49	2.21	Bal.
11	1.32	1.29	0.32	9.21	0.70	0.46	2.23	Bal.
12	1.40	1.29	0.33	9.23	0.73	0.46	1.92	Bal.
13	1.46	1.29	0.34	9.27	0.75	0.45	1.94	Bal.
14	1.50	1.30	0.31	9.20	0.72	0.50	2.04	Bal.
15	1.43	1.29	0.30	9.18	0.72	0.41	2.04	Bal.

**Table 10:** High-Temperature Tensile Test Data. (Note: The red font is used to highlight that fractures consistently occurred in the weld metal (WM) region across all test temperatures).

Temp. (°C)	Dia (mm)	YS (0.2%) (MPa)	UTS (MPa)	Elong. (4D) (%)	RA (%)	Avg. YS (0.2%) (MPa)	Avg. UTS (MPa)	Avg. Elong. (%)	Avg. RA (%)	GB YS (min) (MPa)	GB UTS (min) (MPa)	Fracture Loc.
RT	6.31	513.3	760	18.0	72	514.00	758.33	19.00	69.67	550	800	WM
	6.23	513	757	20.0	67							WM
	6.28	516	758	19.0	70							WM
250 °C	6.27	451.5	591.5	17.5	75.0	453.83	596.17	17.33	75.67			WM
	6.28	455.0	598.5	17.0	76.0							WM
	6.27	455.0	598.5	17.5	76.0							WM
300 °C	6.31	448.0	591.5	18.5	76.0	446.83	591.50	17.83	75.67			WM
	6.26	448.0	591.5	17.0	75.0							WM
	6.29	444.5	591.5	18.0	76.0							WM
350 °C	6.39	441.0	570.5	17.0	72.0	444.50	567.00	17.00	73.33			WM
	6.28	448.0	570.5	17.0	72.0							WM
	6.27	444.5	560.0	17.0	75.0							WM
400 °C	6.29	420.0	542.5	18.0	70.0	417.67	542.50	18.67	73.33			WM
	6.28	416.5	542.5	19.5	75.0							WM
	6.28	416.5	542.5	18.5	75.0							WM
450 °C	6.26	395.5	486.5	19.0	77.0	394.33	493.50	20.83	78.33	420	500	WM
	6.27	395.5	497.0	22.5	80.0							WM
	6.30	392.0	497.0	21.0	78.0							WM
500 °C	6.24	367.5	437.5	21.5	84.0	367.50	435.17	22.17	84.00			WM
	6.30	367.5	430.5	22.0	83.0							WM
	6.29	367.5	437.5	23.0	85.0							WM
550 °C	6.29	315.0	381.5	25.5	87.0	315.00	375.67	24.83	87.33	320	400	WM
	6.27	315.0	371.0	26.5	88.0							WM
	6.27	315.0	374.5	22.5	87.0							WM

**Table 11:** Room Temperature Impact Test Results.

Welding Method	Notch Location	Impact Absorbed Energy KV2 (J)				
		Value 1	Value 2	Value 3	Value 4	Value 5
Manual TIG	WM	-	-	177.7	181.7	192.2
	HAZ	67.1	77.4	66.1	92.0	87.1

## Data Availability

The original contributions presented in this study are included in the article. Further inquiries can be directed to the corresponding author.
